# Continuous particle separation using pressure-driven flow-induced miniaturizing free-flow electrophoresis (PDF-induced μ-FFE)

**DOI:** 10.1038/srep19911

**Published:** 2016-01-28

**Authors:** Hyungkook Jeon, Youngkyu Kim, Geunbae Lim

**Affiliations:** 1 Department of Mechanical Engineering, Pohang University of Science and Technology (POSTECH), San 31, Hyoja-dong, Nam-Gu, Pohang, Gyeongbuk, 790-784, the Republic of Korea

## Abstract

In this paper, we introduce pressure-driven flow-induced miniaturizing free-flow electrophoresis (PDF-induced μ-FFE), a novel continuous separation method. In our separation system, the external flow and electric field are applied to particles, such that particle movement is affected by pressure-driven flow, electroosmosis, and electrophoresis. We then analyzed the hydrodynamic drag force and electrophoretic force applied to the particles in opposite directions. Based on this analysis, micro- and nano-sized particles were separated according to their electrophoretic mobilities with high separation efficiency. Because the separation can be achieved in a simple T-shaped microchannel, without the use of internal electrodes, it offers the advantages of low-cost, simple device fabrication and bubble-free operation, compared with conventional μ-FFE methods. Therefore, we expect the proposed separation method to have a wide range of filtering/separation applications in biochemical analysis.

In biochemical analysis and molecular detection, microfluidic devices have been extensively developed due to their many advantages, including reduced sample and reagent consumption, rapid analysis, and the potential for parallel, automated operation^1^,^2^,^3^. Advances in micro- and nanofabrication techniques have accelerated the growth of microfluidic device development in various application fields, including filtration and separation systems. Continuous-flow separation studies have been performed to optimize micro total analysis systems (μTASs)^1^, based on microstructures^4^, laminar flow^5^, and separation using a non-inertial force field (via dielectrophoretic force^6^, optical force^7^, magnetic force^8^, and acoustic force^9^).

Separation methods based on microstructures and laminar flow have a critical advantage that particles can be continuously separated by only a hydrodynamic stream in the microchannel, without an external force field. However, such hydrodynamic filters have limitations, including poor separation resolution^10^,^11^, a complicated chip design due to microstructures^4^, and limited separation markers^12^. In the case of separation methods using non-inertial force fields, particles can be separated according to various properties such as size, density, magnetic susceptibility, refractive index, and dielectric constant. Despite the versatility in separating particles, these methods have a critical limitation with regard to the separation of nano-sized particles, because the magnitude of most non-inertial forces is proportional to the particle volume. For example, in dielectrophoresis (DEP), it is difficult to sort nanoscale particles due to the electric field decay with distance from the electrode edge, as well as the inverse cubic decay of the DEP force with particle size^13^,^14^.

Electrophoresis separation is recognized as a common technique in biochemistry for separating biomolecules, such as proteins, peptides, DNA, and cells. Because the electrophoretic force does not decay cubically with the particle size, the electrophoresis-based separation techniques maintain high separation resolution at the micro- and nanoscale. Among the various electrophoresis-based separation techniques, miniaturizing free-flow electrophoresis (μ-FFE) has recently received much attention as an electrophoresis-based separation method, due to the advantages of continuous flow separation and microfluidics^15^,^16^,^17^. In μ-FFE, charged samples injected into a thin carrier flow are deflected from the flow by a perpendicular electric field that is formed using internal electrodes; the deflection angle is determined by the competing hydrodynamic drag and electrophoretic forces^18^. The electrophoretic force is proportional to the electrophoretic mobility; thus, charged particles can be separated based on electrophoretic mobility. The advantage offered by μ-FFE includes continuous, high-resolution separation with minimal sample quantities. This suggests the incorporation of μ-FFE as a micro-preparative part of a μTAS system, which is commonly used to separate biomolecules, such as proteins, peptides, DNA, and cells^19^.

Despite excellent performance, μ-FFE has several problems, including a complex fabrication process, gas bubble generation, and Joule heating^15^,^20^. Typically, in the manufacture of μ-FFE devices, the fabrication processes include multi-step etching, soft lithography, anodic bonding, and thermal bonding for integration of the internal electrodes^19^. Gas bubbles are inherently generated at the internal electrodes by water electrolysis and tend to distort separation and decrease the separation efficiency^20^. Research groups have proposed various technological approaches to address this issue, such as using a membrane-like structure^21^,^22^,^23^ or an insulating wall^24^ to block bubble penetration into the separation channel, as well as circulating the flow around the internal electrodes for bubble ventilation^25^,^26^,^27^. Although these methods prevent gas bubble interference, the additional blocking system and the complex ventilation design make the μ-FFE fabrication process more complex and reduce voltage efficiency, with most of the voltage drop occurring in the additional blocking system^17^,^26^. To overcome the limitations of μ-FFE resulting from the internal electrodes, we developed a novel separation method based on pressure-driven flow-induced electrophoresis (PDF-induced μ-FFE).

In our separation system, we apply both pressure-driven flow and an electric field via external electrodes in the parallel direction to the device to manipulate particle movement. The particle movement is determined by the competition between the hydrodynamic drag and the electrophoretic forces, which work in parallel and opposite directions, in which the perpendicular direction is not a factor. The hydrodynamic drag force is induced not only by the applied pressure but also by the electroosmotic flow. Therefore, we quantitatively analyzed particle movement in a separation system affected by pressure-driven flow, electroosmosis, and electrophoresis. The results show that electroosmosis was negligible compared with pressure-driven flow and electrophoresis, when the microchannel surfaces were coated with 1 wt% bovine serum albumin (BSA), a material that is commonly used to prevent non-specific binding of particles. Therefore, certain fluidic and electrical conditions can be used to separate particles that have two electrophoretic mobilities. Particles with lower electrophoretic mobility move along the pressure-driven flow due to the stronger hydrodynamic drag force compared with the electrophoretic force, whereas the second group of particles, with higher electrophoretic mobility, moves in the opposite direction to that of the pressure-driven flow due to the stronger electrophoretic force compared with the hydrodynamic drag force. Thus, using this simple mechanism, micro- and nano-sized particles are separated by the proposed PDF-induced μ-FFE device.

In this study, we analyzed particle movement under the application of pressure-driven flow and an electric field, resulting in the development of a new electrophoresis-based separation device. The developed separation device can also effectively separate nano-sized particles as well as micro-sized ones with high separation efficiency (>97%) because the forces used to manipulate the particles, i.e., the hydrodynamic drag force and the electrophoretic force, are proportional to the particle radius. Because the electric field is directly applied by external electrodes, this method has the advantages of simple, low-cost fabrication, without gas bubble formation or loss of electric field that can occur in the bubble-blocking system. Also, since the electric field is applied in the parallel direction to the fluidic stream, it is possible to separate or filter a target particle from the sample in the opposite direction, which makes it easy to sort and extract the separated two kinds of particles; this is a difference from traditional μ-FFE techniques in which the electric field is applied perpendicular to the stream flow. Although the device is limited to the separation of two particle types in a single step, we expect that the separation method could be widely used as a separation or filtering system for biochemical analysis.

## Results

### Movement of particles under the application of a pressure-driven flow and an electric field

**Figure 1:**
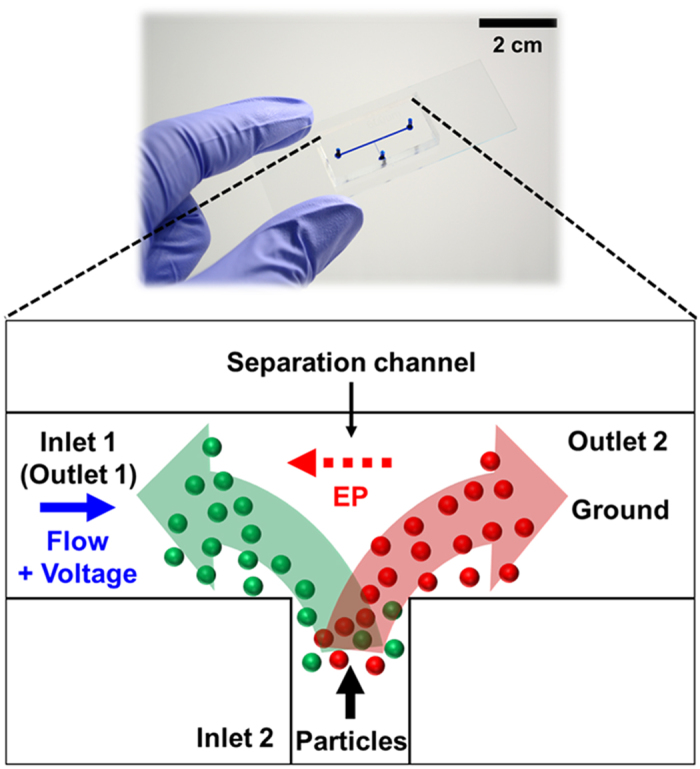
Schematic diagram of the separation device and separation process. (Microchannel: polydimethylsiloxane (PDMS); microchannel height: 45 μm; different colors represent particles having different properties).

Figure 1 shows the layout of the PDF-induced μ-FFE device and a schematic diagram of the separation process. To apply an electric field to the device, anode and cathode electrodes are connected to the metallic syringe tube of Inlet 1 (=Outlet 1) and Outlet 2, respectively. The pressure-driven flow from Inlet 1 moves the particles injected from Inlet 2 along the flow stream to Outlet 2, without the application of an electric field. Under an applied electric field between inlet 1 (=Outlet 1) and Outlet 2, negatively charged particles are affected by an electrophoretic force in the direction directed opposite to that of the flow stream.

**Figure 2:**
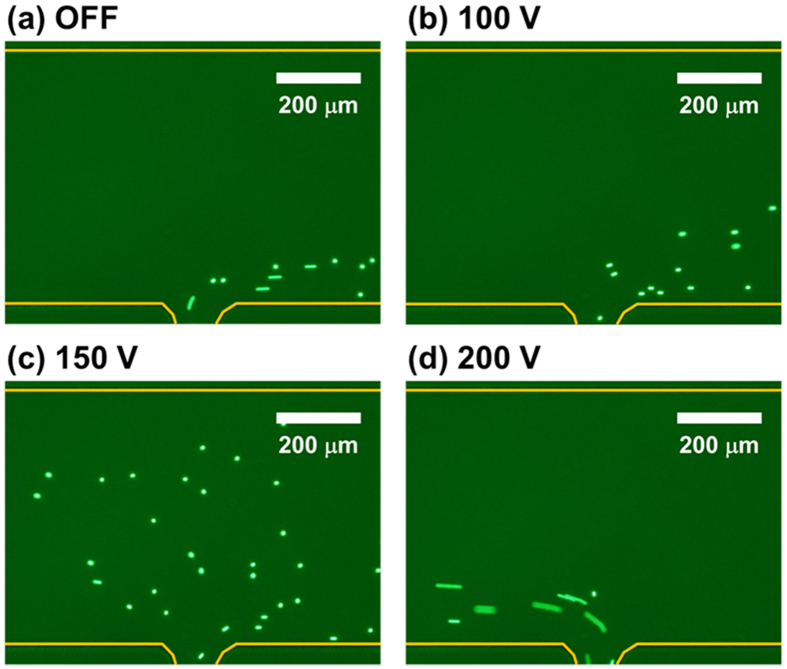
Movement of 4.8-μm particles in the separation channel as a function of the applied voltage: (**a**) off, (**b**) 100 V, (**c**) 150 V, (**d**) 200 V (flow rate of Inlet 1: 400 nL/min; flow rate of Inlet 2: 100 nL/min).

First, we demonstrate the effects of the electrophoretic force on particle movement. Figure 2 shows that the particle movement depends on the strength of the applied voltage. The movement of the particles is determined by the competition between the hydrodynamic drag and the electrophoretic forces. As the applied voltage increases, the particle trajectories are spread over a broader area due to the increased electrophoretic force (Fig. 2a–c). When the applied voltage is higher than the critical voltage (200 V), the particles begin to move against the flow stream due to the higher electrophoretic force compared with the hydrodynamic drag force (Fig. 2d).

### Theoretical analysis of particle movement

Because the electric field is applied to a straight separation channel, the generated electric field is almost uniform. As such, the dielectrophoretic force, a representative force for electrokinetic phenomena arising from nonuniform electric fields^6^, was negligible in this study with regard to its effect on particle separation. We hypothesized that the main influences on particle motion in a straight channel are the hydrodynamic drag force and electrophoretic force.

For the Stokes regime (low Reynolds number regime), the inertial effect is negligible. Therefore,


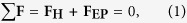


where 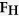 and 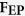 denote the hydrodynamic drag and electrophoretic forces, respectively. For a spherical particle of radius *α* under a direct current (dc) electric field **E**, these forces are expressed as^6^


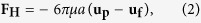



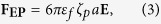


where μ, 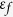, and 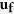 represent the dynamic viscosity, electric permittivity, and velocity of the surrounding flow, respectively, and 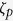 and 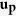 are the surface potential and the velocity of a particle, respectively.

The velocity of the surrounding flow, 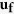, is composed of pressure-driven and electroosmotic flows. The electroosmotic flow is expressed by the Smoluchowski equation^28^, as follows:


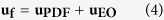



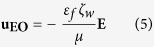


where 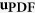, 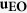, and 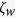 denote the velocities of the pressure-driven and electroosmotic flows and the surface potential of the microchannel, respectively.

We can substitute Eqs (2)–(5), 12, 11, 12 into Eq. (1) and rearrange the equation to solve for 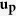:


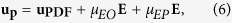


where 
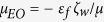
 and 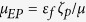 represent the electroosmotic mobility of the microchannel and the electrophoretic mobility of a particle, respectively.

Generally, the surfaces of the microfluidic channel are made from poly(dimethylsiloxane) (PDMS) and glass, and are negatively charged^29^. Therefore, an electric double layer composed of cations forms around the surfaces, and electroosmotic flow is generated in the same direction as the applied electric field. From Eq. (6), negatively charged particles experience oppositely directed electrophoretic and drag forces under these circumstances.

### Experimental measurement of electroosmotic mobility

**Figure 3:**
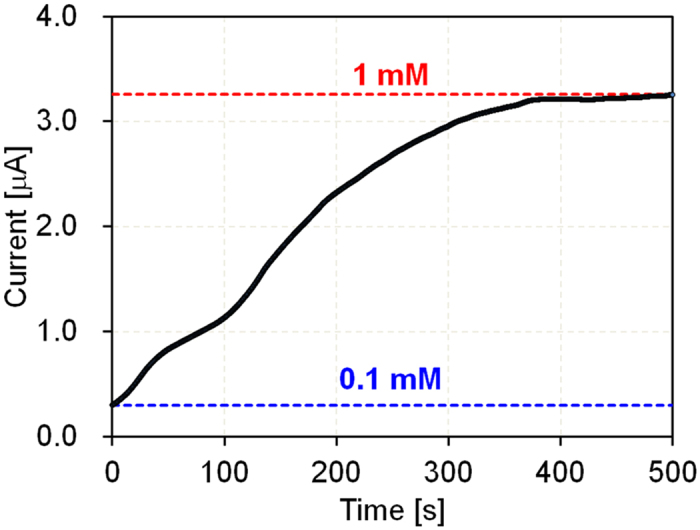
Measurement of electroosmotic mobility based on the current-monitoring method. A PDMS/glass straight microfluidic channel was used (microchannel width: 200 μm; height: 46 μm; length: 2 cm; cathodic reservoir and channel: 0.1-mM DSP; anodic reservoir: 1-mM DSP; applied voltage: 100 V).

The movement of particles is affected by not only electrophoresis but also electroosmosis, as given in Eq. (6). Therefore, to determine the effect of electroosmosis on the movement of particles in the separation channel, we measured the electroosmotic velocity using a current-monitoring method^29^,^30^. A simple, straight PDMS/glass microfluidic device was coated with 1 wt% BSA. An electric field was applied between two reservoirs. The cathodic reservoir and channel were filled with 0.1-mM dibasic sodium phosphate (DSP), and the anodic reservoir was filled with 1-mM DSP. Under an applied voltage of 100 V, the more diluted buffer penetrated the channel due to electroosmotic flow. The increase in current was monitored until the channel was completely filled (Fig. 3). The electroosmotic mobility, 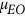, was calculated using the following equation:


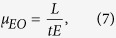


where *L, t*, and *E* represent the channel length (2 cm), the time required to achieve a constant current, and the electric field strength (100 V per 2 cm), respectively. The time required to achieve a constant current was ~400 s, as shown in Fig. 3. From Eq. (7), the electroosmotic mobility was ~1 × 10^−4^ cm^2^ V^−1^s^−1^.

### Effect of electrophoretic mobility on particle movement

**Figure 4:**
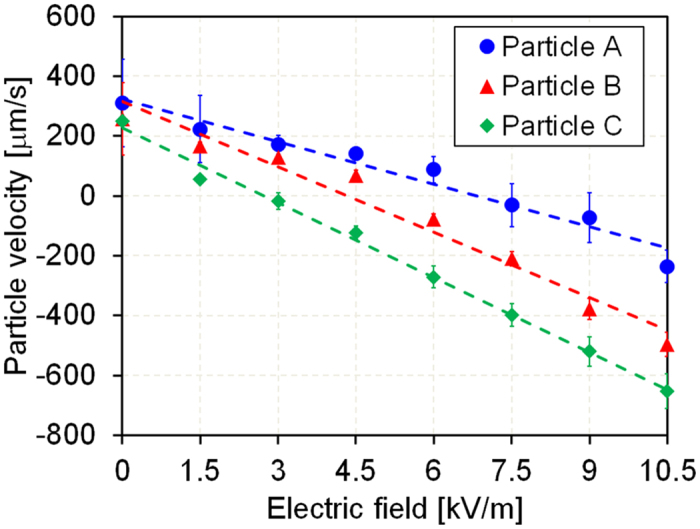
Particle velocity dependence on the applied electric field. Particles (A, B, and C) had electrophoretic mobilities of −4.35 × 10^−4^, 6.18 − 10^−4^ and −8.44 × 10^−4^ cm^2^∙V^−1^ s^−1^ and diameters of 4.8, 9.9, and 10 μm, respectively (microchannel width: 200 μm; height: 46 μm; length: 2 cm; and flow rate: 200 nL/min).

**Figure 5:**
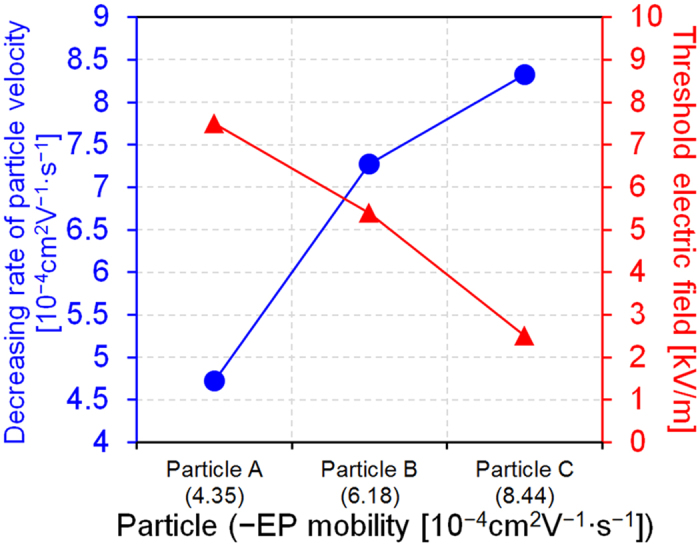
Decreasing rate/particle velocity with increasing electric field strength and the threshold electric field initiating particle movement against the flow stream; particles (A, B, and C) exhibited electrophoretic mobilities of −4.35 × 10^−4^, 6.18 × 10^−4^, and −8.44 × 10^−4^ cm^2^ V^−1^ s^−1^, respectively.

To demonstrate the effect of electrophoretic mobility on particle movement, we measured the velocities of particles of different sizes and their electrophoretic mobility under an applied electric field, given a fixed flow rate in a simple, straight channel. Electrophoretic mobility of particles in a buffer solution, 1-mM DSP with a pH level of 7.1, was measured using a zeta potential analyzer (Table 1), and particle velocity was measured using particle image velocimetry (PIV) software. Under an applied electric field, an electrophoretic force and hydrodynamic drag force from electroosmotic flow were applied to particles in the opposite direction, given that the particles used were negatively charged. Because the absolute value of electrophoretic mobility is higher than that of electroosmotic mobility (~1 × 10^−4^ cm^2^V^−1^s^−1^), the particles move in the direction of electrophoretic force according to Eq. (6), in the absence of pressure-driven flow. Therefore, when electrophoretic force is applied to the particles in a direction opposite to that of the drag force due to pressure-driven flow, particle velocity decreases linearly with an increased electric field, as shown in Fig. 4. When the applied electric field is higher than a certain threshold electric field, the electrophoretic force becomes stronger than the hydrodynamic drag force, and particles begin to move against the flow stream. Under circumstances in which electroosmosis can be neglected, the particle deceleration with an increasing electric field represents the experimental electrophoretic mobility. Therefore, particles with a higher electrophoretic mobility have a lower threshold electric field when the flow stream is constant. In summary, as shown in Fig. 5, our results show that the decrease in the rate of particle velocity was nearly the same as the measured electrophoretic mobility, and the threshold electric field decreased as the absolute value of the electrophoretic mobility increased.

### Separation of micro- and nano-sized particles

**Figure 6:**
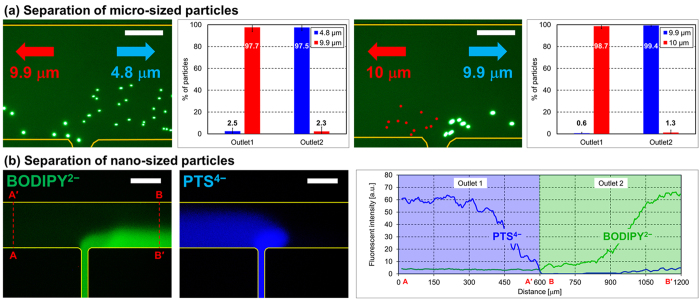
Separation of micro- and nano-sized particles depending on electrophoretic mobility. (**a**) Diameters of 4.8, 9.9, and 10 μm corresponded to electrophoretic mobilities of −4.35 × 10^−4^, −6.18 × 10^−4^, and −8.44 × 10^−4^ cm^2^∙V^−1^∙s^−1^, respectively (snapshot with image processing to represent nonfluorescent 10-μm glass particles; flow rate of Inlet 1: 400 nL/min; flow rate of Inlet 2: 100 nL/min; applied voltage: 130 V (left) and 80 V (right); scale bar: 200 μm). Graphs represent quantitative analysis showing the ratio of particles moving to each outlet. (**b**) Molecule dyes: BODIPY^2–^ and PTS^4–^: green and blue fluorescence expression, respectively (snapshot with high contrast; flow rate of Inlet 1: 300 nL/min; flow rate of Inlet 2: 30 nL/min; applied voltage: 120 V; scale bar: 400 μm). Graphs represent the fluorescent intensities of each molecule dye at the outlets (along the line, A-A′-B-B′).

Electrophoresis can be used to separate a wide range of particle sizes, from the micro- to the nano-scale. Based on the change in the threshold electric field with electrophoretic mobility, we attempted to separate micro- and nano-sized particles using a simple T-shaped microfluidic device (height: 40 μm). Figure 6a shows the separation of microparticles having different electrophoretic mobilities (see the Supplementary Videos 1 and 2). For a given flow rate and voltage (Inlet 1: 400 nL/min, Inlet 2: 100 nL/min, voltage: 140 V), 9.9-μm particles moved against the flow stream, due to higher electrophoretic mobility, whereas 4.8-μm particles moved with the flow stream; the two microparticles were continuously separated. Despite being almost the same size, 9.9-μm and 10-μm particles were separated in the same manner. We measured the separation efficiency by counting the number of particles moving to each outlet (n > 5). As shown in Fig. 6a, the micro-sized particles were separated with high separation efficiency (>97%). Figure 6b shows the separation of dye molecules having a size on the order of 1 nm: 4,4-difluoro-1,3,5,7,8-pentamethyl-4-bora-3a,4a-diaza-s-indacene-2,6-disulfonic acid, disodium salt (BODIPY^2–^, Invitrogen), and 1,3,6,8-pyrene tetrasulfonic acid (PTS^4–^, Sigma-Aldrich), all with different electrophoretic mobilities, PTS^4–^ > BODIPY^2–^ 31. For a given flow rate and electric field strength across the separation channel, we observed that PTS^4–^ moved against the flow stream, while BODIPY^2–^ moved with the flow stream; the two nanoparticles were separated continuously in the same manner as with the separation of microparticles. A graph in Fig. 6b represents the separation of the molecule dyes quantitatively by analyzing fluorescent intensities of each molecule dye at outlets (along the line, A-A′-B-B′).

## Discussion

When applying both pressure-driven flow and an electric field to the separation channel, the motion of particles in the channel is determined by the hydrodynamic drag force and electrophoretic force. The hydrodynamic drag force is affected by pressure-driven flow and electroosmotic flow. From Eq. (6), the electrophoretic force and the drag force resulting from electroosmosis are proportional to the electric field and are applied to negatively charged particles in opposite directions.

To analyze particle motion, it is important to compare the effects of electroosmosis and electrophoresis. We experimentally measured electroosmotic mobility in a PDMS/glass microfluidic channel coated with 1 wt% BSA; the electroosmotic mobility of the channel was lower than the electrophoretic mobility of the particles. Therefore, under an applied electric field without an external flow stream, negatively charged particles moved in the direction of electrophoresis (opposite to the electric field direction). Under a uniform external flow stream, we were able to control the particle motion by changing the external electric field, as well as separate particles depending on their electrophoretic mobilities. As explained above, separation was possible because the electroosmotic mobility of the microchannel was lower than electrophoretic mobility of the particles. In the case of separation devices with channels coated with 1 wt% Pluronic, a representative coating material for preventing non-specific binding similar to BSA, it was difficult to control particle motion to separate the particles. The electroosmotic mobility results for the device coated with 1 wt% Pluronic show that the electroosmotic mobility was more than twice that in the device with 1 wt% BSA (see the Supplementary Note and Fig. S1). When the electroosmotic mobility is high and comparable to the electrophoretic mobility, the drag force due to the electroosmotic flow is strongly applied to the particles in the direction opposite to that of the electrophoretic force, which makes it difficult to control particle motion using an external electric field and increases the applied voltage needed for particle separation. Therefore, it is very important to reduce the electroosmotic mobility of the separation device to increase separation efficiency.

Because the pressure-driven flow formed is parabolic, the hydrodynamic drag force applied to the particles varies depending on their position in the channel, which results in variation in the velocity of particles, as shown in the error bars of Fig. 4, and broadened particle dispersion, as shown in Fig. 5. Three-dimensional (3-D) flow focusing will be adapted to the device for more uniform application of the drag force on the particles; we expect this to reduce the dispersion and improve separation resolution. Also, in case of nanoparticles, due to the small characteristic length and the high mass diffusivity, diffusion transport becomes significantly strong compared to convection transport of flow stream, which results in low Peclet number (Pe < 1); the Peclet number indicates the ratio of convection to diffusion transport^32^. Due to the strong diffusion transport, the dispersion and diffusion of particles are observed more intensely in separation of nanoparticles than microparticles as shown in Fig. 6, which can affect the separation efficiency significantly. However, in our separation system, we can reduce the diffusion effect by strengthening both flow stream and electric field because the motion of particles is determined by the competition of hydrodynamic force and electrophoretic force, although the stronger flow stream and electric field would require higher power consumption. Therefore, in our separation system, it is important to control strengths of the flow stream and electric field considering the diffusion effect, separation efficiency, and power consumption.

The charge of many biochemical analytes, including proteins, is highly dependent on the local pH level; the change in the surface charge affects the electro-migration of the analytes in the presence of an electric field^20^. In the μ-FFE system, therefore, maintaining the pH level of the carrier medium is very important in terms of the separation stability and efficiency. The variation in pH level is generated mainly near the electrodes, due to electrochemical reactions at the electrode surface under an applied electric field. The variation in pH level near the electrodes can be controlled by the convective flow around the electrodes. Dietrich *et al*.^20^ demonstrated that the region available for separation is limited by the pH variation, and area of the region varies depending on the flow velocity and the applied electric field in the μ-FFE system. They measured the propagation speed of the H + enriched region (pH < 6) from an anode using the pH-dependent fluorescence of fluorescein, which practically disappeared below a pH of 6. The speed was determined to be ~400 μm/s using a flow velocity of 2 mm/s and an electric field of 20 kV/m applied in a perpendicular direction with respect to the flow stream using internal electrodes. In our PDF-induced μ-FFE system, pH variation can be generated near the external electrodes. However, the flow stream (10~20 mm/s) applied in the parallel direction to the electric field (5 kV/m) can decrease the variation in pH near the electrodes in our device; as the electrolyte near the electrodes is continuously exchanged by the incoming flow of new electrolyte with the relatively high flow stream, while in conventional μ-FFE systems, the pH of electrolyte near the electrodes accumulatively changes because the flow stream is applied along the plate-typed electrodes. Furthermore, because the region of sample separation is far from the electrodes, the effect of pH variation on separation performance is negligible.

In our PDF-induced μ-FFE device, Inlet 1 and Outlet 1 work with the same reservoir and tube. Therefore, it may be difficult to collect the separated particles of Outlet 1 continuously, compared with Outlet 2. However, because the electrode of Outlet 1 is located at the reservoir through the hole of the syringe tube, the separated particles of Outlet 1 were collected at the reservoir, thus, allowing extraction of the separated particles from the reservoir for biochemical analysis. To overcome the limitations of continuous collection at Outlet 1, we plan to re-design our device to break down Inlet 1 (=Outlet 1) into two separate parts where one channel is only for applying flow stream (Inlet 1) and the other for applying electric field (Outlet 1) by installing an additional channel; thus, particles separated in the T-shaped region can be continuously collected in Outlet 1 and Outlet 2, respectively. In this study, we focused on developing a new-type of μ-FFE device to resolve the critical limitations of traditional μ-FFE devices. Based on the current results, we will re-design the separation device and use it for continuous separation of biochemical samples.

In summary, we developed a novel continuous separation method that uses PDF-induced μ-FFE. Experiments were performed to quantitatively analyze particle movement in response to pressure-driven flow, electroosmosis, and electrophoresis, to verify the separation principles. In our separation system, electrophoresis is dominant in particle movement compared with electroosmosis. The threshold electric field at which the particles begin to move against the surrounding flow stream decreases as the absolute value of the electrophoretic mobility increases. Using this simple mechanism, we separate micro-and nano-sized particles continuously depending on their electrophoretic mobility with high separation efficiency (>97%). Because the electric field is applied in the parallel direction to the fluidic stream, it is possible to separate or filter a target particle from the sample in the opposite direction, which makes it easy to sort and extract the separated two kinds of particles. The T-shaped separation device is fabricated using a one-step photolithography process, and an electric field is applied to the separation device by external electrodes. Therefore, this separation method has the advantage of simple, low-cost fabrication without loss of voltage or gas bubble formation, a serious issue in conventional electrophoresis-based separation methods. Although this device is limited to separation of only two kinds of particles in a single step, we anticipate that it will be useful in separation and filtering systems for biochemical analysis. Additionally, the quantitative analysis of electrokinetic phenomena presented in this work, including electroosmosis and electrophoresis, should be applicable to the development of various microfluidic devices.

## Methods

We fabricated a polydimethylsiloxane (PDMS) microfluidic device using conventional photolithography (microchannel height: 45 μm, separation channel width: 600 μm, separation channel length: 2 cm)^33^. The fabricated microfluidic device contained T-shaped microchannels, as shown in Fig. 1. The PDMS microfluidic layer was bonded to a slide-glass by oxygen plasma treatment. The microchannels were surrounded by three PDMS surfaces and one glass surface^15^.

In our experiments, we used fluorescent polystyrene particles and glass particles (ThermoScientific Corp. and Invitrogen, respectively) of various sizes and electrophoretic mobilities; however, all of the mobilities had a negative value (see Table 1). The molecular dyes used, 4,4-difluoro-1,3,5,7,8-pentamethyl-4-bora-3a,4a-diaza-s-indacene-2,6-disulfonic acid, disodium salt (BODIPY^2–^, Invitrogen), and 1,3,6,8-pyrene tetrasulfonic acid (PTS^4–^, Sigma-Aldrich), were of similar size (order of 1 nm), but different electrophoretic mobilities, PTS^4–^>BODIPY^2–^ 31.

We used 1-mM dibasic sodium phosphate (DSP) solution having a pH level of 7.1 as a buffer solution. A 1 wt% BSA (A9647, Sigma Aldrich) solution was used to coat the channel surfaces to prevent non-specific binding of particles. An electric field was applied to the separation device using a source measurement unit (B2902A, Keysight). The flow rates of inlets 1 and 2 were regulated using two syringe pumps (Pump 11 Elite, Harvard Corp.). An inverted fluorescence microscope (IX71, Olympus) and a charge-coupled device (CCD) camera (DP72, Olympus) were used to observe the trajectories of the fluorescent particles and collect images from the device.

For electric field application, anode and cathode electrodes were connected to the syringe tube of Inlet 1 (=Outlet 1) and Outlet 2 reservoir, respectively. Specifically, to apply both voltage and flow stream to the Inlet 1 (=Outlet 1) simultaneously, a Pt electrode was inserted in the syringe tube through a cut hole that was sealed with epoxy afterwards. An electric field was applied across the separation channel (length: 2 cm). In the absence of an applied electric field, using a flow-focusing method^34^, particles injected from Inlet 2 moved to Outlet 2 via the buffer solution flow of Inlet 1; no particles were injected from Inlet 1.

## Additional Information

**How to cite this article**: Jeon, H. *et al*. Continuous particle separation using pressure-driven flow-induced miniaturizing free-flow electrophoresis (PDF-induced µ-FFE). *Sci. Rep.* **6**, 19911; doi: 10.1038/srep19911 (2016).

## Supplementary Material

Supplementary Video 1

Supplementary Video 2

Supplementary Note

## Figures and Tables

**Table 1:** Electrophoretic mobility for given polystyrene and glass particle diameters.

**Particle diameter (**μ**m)**	**Electrophoretic mobility (10^−4^ cm^2^V^−1^s^−1^)**
4.8 (polystyrene)	−4.35
9.9 (polystyrene)	−6.18
10 (glass)	−8.44

The mobilities of particles diluted in 1-mM DSP solution were measured by a zeta potential analyzer (Zetasizer Nano Z, Malvern).
