# Supplementary material for: Continuous particle separation using pressure-driven flow-induced miniaturizing free-flow electrophoresis (PDF-induced μ-FFE)
Source: Sci Rep. 2016 Jan 28;6:19911. doi: 10.1038/srep19911 (PMC4730231; doi:10.1038/srep19911)
Supplement: Supplementary Note [file srep19911-s3.doc]

Continuous particle separation using pressure-driven flow-induced miniaturizing free-flow electrophoresis (PDF-induced μ-FFE)

Hyungkook Jeon, ^†^ Youngkyu Kim, ^†^ and Geunbae Lim*^,†^

^†^Department of Mechanical Engineering, Pohang University of Science and Technology (POSTECH), San 31, Hyoja-dong, Nam-Gu, Pohang, Gyeongbuk, 790-784, the Republic of Korea

**Corresponding Author**

*Geunbae Lim, Department of Mechanical Engineering, Pohang University of Science and Technology, San 31, Hyoja-dong, Nam-Gu, Pohang, Gyeongbuk, 790-784, the Republic of Korea, Tel. +82-54-279-2186, Fax +82-54-279-0479, e-mail: limmems@postech.ac.kr

**Supplementary Note**

**Measurement of electroosmotic mobility depending on coating materials**

To demonstrate the effects of coating materials on electroosmotic mobility, we measured the electroosmotic mobility by using current-monitoring method^1,2^ depending on coating materials, 1 wt% BSA (A9647, Sigma Aldrich) and 1 wt% pluronic F68 (P1300, Sigma Aldrich) which are representative coating materials to prevent non-specific binding. We used a simple straight poly(dimethylsiloxane) (PDMS)/glass microfluidic device coated with 1 wt% BSA and pluronic and applied the electric field between the two reservoirs. The cathodic reservoir and the channel were filled with 0.1-mM dibasic sodium phosphate (DSP), whereas the anodic reservoir was filled with 5-mM DSP. Under applying voltage (100 V), the more diluted buffer solution is penetrating into the channel due to electroosmotic flow, showing an increase in current until the channel is completely filled with the more diluted buffer solution (Figure S1). The electroosmotic mobility, $\mu_{EO}$, was calculated by the following equation,

$$\mu_{EO}=\frac{L}{tE},$$

where $L$, $t$ and $E$ represent the channel length (2 cm), required time to reach a constant current and the strength of the electric field (100 V/ 2 cm), respectively.


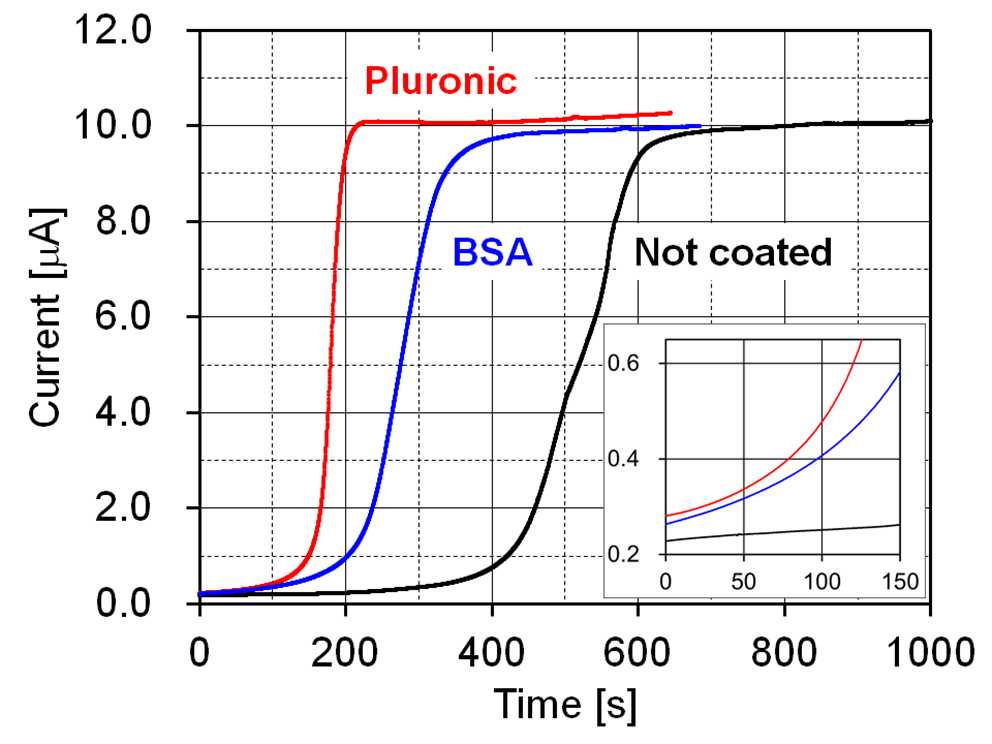


**Figure S1 | Measurement of electroosmotic mobility based on the current-monitoring method depending on coating materials;** 1 wt% BSA (blue), 1 wt% pluronic (red) and not coated (black, control), A PDMS/glass straight microfluidic channel was used (microchannel width: 200 μm; height: 46 μm; length: 2 cm; the cathodic reservoir and the channel: 0.1-mM DSP; the anodic reservoir: 50-mM DSP; applied voltage: 100 V).

As shown in Figure S1, the required times of the coated channels are shorter than the time of the not coated channels. Especially, the required time of the pluronic coated channel is less than half of BSA coated channel. Because the electroosmotic mobility is inversely proportional to the required time, the electroosmotic mobility inside the pluronic coated channel is higher than twice the mobility inside the BSA coated channel. As a result, in case of the pluronic coated separation device, the drag force due to the electroosmotic flow is strongly applied to the particles in the opposite direction of the electrophoretic force, which makes it difficult to control particle motion using an external electric field and increases the applied voltage for particles separation. Therefore, it is very important to reduce the electroosmotic mobility of the separation device to increase separation efficiency.

**References**

1. Spehar, A. M. *et al.* Electrokinetic characterization of poly(dimethylsiloxane) microchannels. *Electrophoresis* **24,** 3674–3678 (2003).

2. Huang, X., Gordon, M. J. & Zare, R. N. Current-monitoring method for measuring the electroosmotic flow rate in capillary zone electrophoresis. *Anal. Chem.* **60**, 1837–1838 (1988).
